# Task-Oriented Semantic Feature Transmission for Robust EEG Motor Imagery Decoding Under Additive White Gaussian Noise

**DOI:** 10.3390/s26185728

**Published:** 2026-09-09

**Authors:** Hossein Ahmadi, Luca Mesin

**Affiliations:** Department of Electronics and Telecommunications, Politecnico di Torino, 10129 Turin, Italy; luca.mesin@polito.it

**Keywords:** electroencephalography, brain–computer interface, motor imagery, semantic communication, task-oriented communication, filter bank common spatial pattern, additive white Gaussian noise, remote classification

## Abstract

Remote electroencephalography (EEG) systems require compact representations that remain useful when communication noise corrupts the transmitted message. We evaluated whether task-oriented residual refinement of filter bank common spatial pattern (FBCSP) features compressed by principal component analysis (PCA) improves four-class motor imagery (MI) decoding without increasing the transmitted dimension. The BNCI2014-001 dataset was assessed in nine subjects using bidirectional subject-specific cross-session evaluation. All methods transmitted K∈{16,32,64} power-normalized real values through additive white Gaussian noise (AWGN) at seven signal-to-noise ratios (SNRs) and a noise-free reference. Balanced accuracy was averaged over 20 paired noise realizations per noisy condition, and paired subject-level differences were evaluated with exact joint sign-flip max-|t| inference. At K=32, the proposed method achieved 41.10%, 49.46%, and 56.15% balanced accuracy at −10, −5, and 0 dB, compared with 38.63%, 46.28%, and 53.26% for conventional FBCSP–PCA transmission. Ten of the 24 semantic-versus-conventional comparisons were significant after family-wise max-|t| correction, including all nine comparisons at −10, −5, and 0 dB. Receiver-only controls closely reproduced conventional performance at all three message dimensions, whereas alternative loss weights, uniform-SNR training and selection, and removal of the 0 dB/noise-free reference-condition guard retained positive low-SNR gains. Overall, baseline-preserving task-oriented refinement improved MI decision robustness under severe AWGN without increasing the number of transmitted values.

## 1. Introduction

In an engineered sensing system, recorded samples acquire practical value through the decision or action they enable. Classical communication theory established how a signal generated at one point can be represented and recovered reliably at another, largely without assigning value to the meaning of the transmitted content [1]. In many sensing systems, however, the measured samples are not the final objective; they are an intermediate description from which a receiver must infer a state, recognize an event, or select an action. If the receiver only needs a particular decision, reproducing every source sample with equal fidelity may be neither necessary nor the most effective use of a constrained message. This observation motivates semantic and task-oriented communication, in which the transmitted representation is judged by its usefulness for the receiver’s goal rather than solely by symbol-level or waveform-level similarity [2,3]. For such applications, the design question can therefore shift from how accurately can the source be reconstructed? to which information must survive the link for the intended decision to remain correct? Throughout this article, sensing denotes instrumented acquisition of physiological signals and does not refer to biological sensory processing.

Electroencephalography (EEG) provides a compelling setting for this question. EEG non-invasively measures electrical potentials at the scalp with high temporal resolution, and brain–computer interfaces (BCIs) use these recordings to create pathways for communication and control that do not depend on conventional muscular output [4,5]. Motor imagery (MI), in which a person imagines a movement without executing it, is one of the principal EEG–BCI paradigms. The present problem therefore concerns transmission of scalp-EEG measurements for a predefined BCI decision. MI-discriminative information is distributed across electrodes, frequency ranges, and time, while the measured signals are affected by low signal amplitudes, background activity, artifacts, and substantial variation between people and recording sessions [5,6]. Wearable and wireless EEG systems permit acquisition away from a fixed laboratory workstation and can separate the sensing device from the computing resource that interprets its data [7]. Such separation creates a communication problem in addition to the decoding problem: the acquisition side must construct a fixed-dimensional representation from each EEG trial, the communication link may disturb that representation, and a classifier at the remote side must still identify the intended MI class. When the communication signal-to-noise ratio (SNR) is low, the added disturbance is large relative to the transmitted signal, so information that supports a reliable local decision may not remain equally reliable after transmission.

Established MI pipelines provide strong foundations for constructing compact EEG messages. Common spatial patterns (CSP) identify weighted combinations of electrodes whose variances discriminate between imagined movements [8]. The filter bank common spatial pattern (FBCSP) method extends this principle across multiple frequency bands and remains an important reference for MI decoding [5,9,10]. Its features can be further reduced by principal component analysis (PCA) to a prescribed dimension. In parallel, convolutional networks and compact architectures such as EEGNet have demonstrated that temporal and spatial EEG representations can be learned directly from data [11,12]. Recent convolutional–transformer models also combine local EEG features with longer-range temporal dependencies [13,14]. These advances have primarily addressed representation quality when acquisition and decision making form a local processing chain. A compact representation that separates MI classes without channel noise is not necessarily arranged to preserve that separation under additive disturbance. Training a remote classifier on noisy features can adapt its decision boundaries, but it cannot change the message constructed at the acquisition side.

Learned EEG representations introduce a further design choice: which information should compression preserve? Autoencoders retain information needed to reconstruct the input waveform, whereas supervised decoders emphasize variations predictive of the task label; suitably designed autoencoder features can nevertheless support MI decoding [15]. Compact convolutional networks, large-scale pretrained encoders, and recent task-independent and topographic approaches have broadened EEG representation learning beyond predetermined handcrafted descriptors [12,16,17,18,19,20]. However, waveform reconstruction and task preservation are not equivalent: a component can contribute substantially to reconstruction error while providing little class information, whereas a low-energy pattern may be essential for classification [21]. These advances therefore do not by themselves determine which representation should be transmitted through a noisy link under a fixed message size.

Task-oriented semantic communication addresses this problem by constructing a message according to its usefulness for the receiver task rather than solely according to source-reconstruction fidelity. Differentiable channel models permit message construction and receiver decision-making to be optimized jointly under the disturbances expected during transmission [22,23,24,25,26]. Here, semantic refers specifically to information optimized for MI classification; it does not imply linguistic meaning, recovery of thought content, or a neurophysiological claim about how the brain represents semantics. Neural applications include semantic communication for multi-brain robot control [27], cross-modal visual-perception communication in the EidetiCom preprint [28], and task-agnostic EEG representations evaluated under uncoded noisy links across several cognitive tasks [29]. Robustness and security have also been studied for EEG-based brain-to-brain communication under adversarial perturbations [30]. Together, these studies establish an emerging connection between neural decoding and communication-aware representation design.

The remaining question is whether a strong conventional MI representation can be refined, rather than replaced, to improve remote decisions after noisy transmission. A controlled answer requires matched message dimension and power, exclusion of the held-out test session from subject-level preprocessing and model selection, receiver-only controls separating message refinement from classifier retraining, and sensitivity analyses of the loss and selection rule. Prior studies do not examine this combination for baseline-preserving FBCSP–PCA refinement under matched real-valued channel uses. The architectural element is intentionally lightweight; the contribution lies in the controlled message-level comparison and in testing whether a strong conventional MI representation can be refined without sacrificing its established starting point.

We therefore formulate MI decoding as remote classification of a compact vector transmitted through an idealized analog additive white Gaussian noise (AWGN) link, with *K* denoting real-valued channel uses rather than bits or a complete wireless protocol. The proposed transmitter learns a bounded residual correction to FBCSP–PCA features, initialized to reproduce and constrained to remain near the conventional system. We compare it with conventional FBCSP–PCA transmission, an exploratory reconstruction-oriented autoencoder, a supervised filter-bank EEGNet transmitter, and receiver-only controls under matched message dimensions and AWGN conditions.

The main contributions of this study are as follows:

1. We formulate and evaluate a remote EEG classification framework in which alternative representations are compared in terms of downstream MI classification while transmitting the same number of power-normalized real values through the same noisy link.
2. We introduce a baseline-preserving semantic transmitter that learns a bounded, task-oriented residual refinement of FBCSP–PCA features, retaining the established conventional representation as its starting point rather than replacing it with an unconstrained latent space.
3. We compare the proposed method with conventional feature transmission, an exploratory autoencoder-based reconstruction comparator, a supervised filter-bank EEGNet transmitter, and receiver-only controls at every communication load. These comparisons separate changes to the message from classifier-only training and place the baseline-preserving design alongside two independently learned message representations.
4. We evaluate the approaches using subject-specific bidirectional cross-session testing, matched numbers of power-normalized real-valued channel uses under the adopted AWGN abstraction, paired channel realizations, exact subject-level family-wise inference, and sensitivity analyses of loss weighting, model selection, subject-level robustness, and computational cost.

Section 2, Section 3, Section 4 and Section 5 respectively present the methods, results, interpretation and limitations, and conclusions.

## 2. Materials and Methods

### 2.1. EEG Dataset

The study was conducted on BNCI2014-001, also known as BCI Competition IV Dataset 2a [31,32,33,34]. The public benchmark contains recordings from nine subjects, and all nine were included; the sample size was therefore determined by the complete dataset rather than by a subject-selection rule. Participants performed four MI tasks: imagining movements of the left hand, the right hand, both feet, or the tongue. At the start of each trial (0 s), a fixation cross appeared together with a brief acoustic warning. At 2 s, a directional cue was displayed for 1.25 s and instructed the participant which imagery task to perform; imagery continued until the fixation cross disappeared at 6 s, followed by a short break. No online feedback was presented. Each subject completed two sessions on different days, with six runs of 48 class-balanced trials per session.

The released continuous signals were acquired with a BrainAmp MR plus amplifier from 22 monopolar Ag/AgCl scalp electrodes arranged at approximately 3.5 cm interelectrode distances, with the left mastoid as reference and the right mastoid as ground. The provider-reported amplifier sensitivity was 100 μV. During acquisition, the signals were band-pass filtered between 0.5 and 100 Hz, notch filtered at 50 Hz, and sampled at 250 samples/s. Thus, 250 Hz is the temporal sampling rate, not the analyzed EEG bandwidth. The three accompanying electrooculographic (EOG) channels were not used. The complete dataset contained 576 trials per subject and 5184 trials before trials marked as artifactual by the provider were removed. Participant-level age, sex, handedness, and hemispheric-dominance information are not supplied with the public release and therefore could not be included as explanatory variables.

The two sessions enabled cross-session evaluation, in which a system fitted using one recording day was evaluated on the other. The following subsections define the remote classification problem and then describe the transmitted representations, data preparation, model training, and evaluation.

### 2.2. Remote EEG Classification as a Communication Problem

We considered a situation in which EEG is recorded at one location, whereas the final MI decision is made at another. For example, a wearable EEG device may collect a trial near the user, while a separate computer performs the classification. Sending the complete trial would require transferring thousands of EEG samples for every decision. The acquisition side therefore replaces the trial with a much shorter numerical message and passes it to the remote processing side. The scientific question is whether the construction of this message determines how reliably the remote computer can identify the intended MI class when the transferred values are disturbed by noise. In this study, communication refers specifically to this transfer of information between the two sides.

The system contains three consecutive stages. First, a software block at the acquisition side converts one EEG trial into a vector containing *K* real numbers. We call this algorithm the transmitter, and its *K*-dimensional output is the message for that trial. Second, the vector passes through an abstract link between the acquisition and remote sides, termed the communication channel. This must not be confused with an EEG channel: an EEG channel is the signal measured by one electrode, whereas the communication channel is the path over which the message is transferred. Third, a software block at the remote side receives the vector and assigns it to one of the four MI classes. We call this block the receiver. Thus, transmission denotes the passage of one message vector from the transmitter to the receiver for an EEG trial. The transmitter and receiver are software processing blocks rather than hardware radio components, and no switching technology is modeled. The study begins with the released continuous EEG recordings and evaluates how trial-level numerical representations behave after the specified communication disturbance.

**Figure 1:**
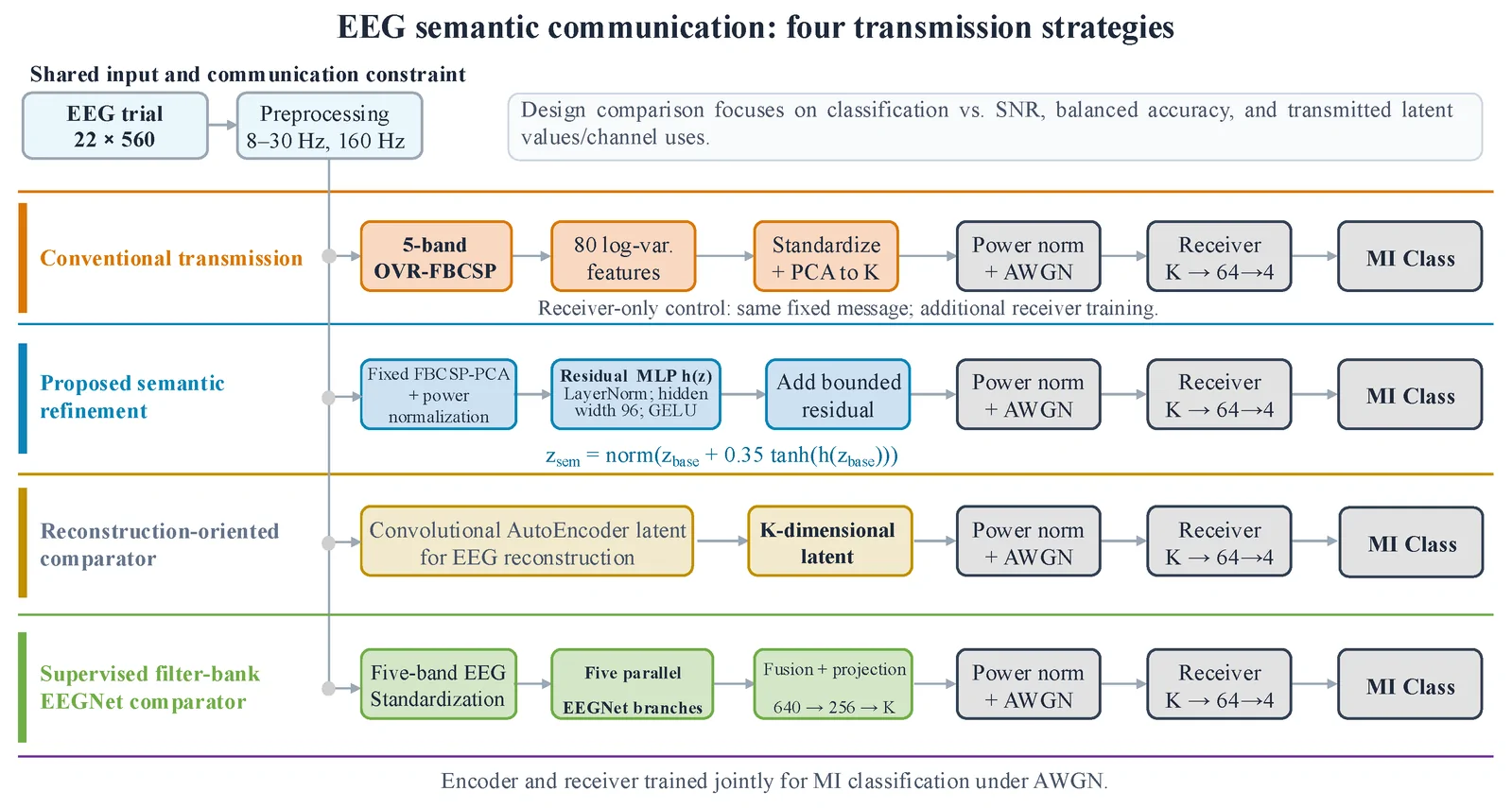
Remote MI-classification framework and four message representations. All methods transmit *K* power-normalized real values through AWGN; the receiver-only control retains the conventional message. OVR, one-versus-rest; MLP, multilayer perceptron; GELU, Gaussian error linear unit.

Figure 1 summarizes this trial-level processing chain and the principal ways in which the message is constructed.

We represent one EEG trial by a real-valued matrix $X\in R^{C\times T}$, where R is the set of real numbers, *C* is the number of EEG electrodes, and *T* is the number of temporal samples. The number of values to be transmitted is denoted by *K*, and f(·) denotes the representation method operating at the acquisition side. The transmitter output is

(1) $$z=f\left(X\right),\;z\in R^{K}.$$

The value *K* is the communication load considered in this study, with K∈{16,32,64}. In the adopted real-valued channel model, each transmitted scalar occupies one real-valued channel use; consequently, a *K*-dimensional vector requires *K* such channel uses. To avoid confusion with the 22 EEG electrode channels, the results are reported primarily as thenumber of transmitted values.

The numerical scale of a transmitted vector determines the relative effect of a fixed noise level. Without normalization, a method could obtain an artificial advantage by producing values with larger amplitudes. We denote the rescaled vector by $\tilde{z}$ and apply per-vector power normalization:

(2) $$\tilde{z}=\sqrt{K}\frac{z}{\max\left({∥z∥}_{2},\varepsilon \right)},\;\varepsilon =10^{-8},$$

where ${∥\cdot ∥}_{2}$ is the Euclidean norm and ε prevents numerical instability when the norm is zero or very small. For ${∥z∥}_{2}\geq \varepsilon$, Equation (2) makes the mean squared value of the *K* transmitted numbers equal to one. The same normalization is applied to every method before transmission.

The communication link is represented by an AWGN model. We denote the added noise by n, its variance by $\sigma_{n}^{2}$, and the vector available at the remote side by r. The communication model is

(3) $$r=\tilde{z}+n,\;n∼N\left(0,\sigma_{n}^{2}I_{K}\right),$$

where $I_{K}$ is the K×K identity matrix. The SNR controls the size of the disturbance relative to the normalized transmitted vector. A high SNR corresponds to weak corruption, whereas a low SNR corresponds to strong corruption. SNR is expressed in decibels (dB). Because Equation (2) sets the mean squared transmitted value to one, the noise variance is

(4) $$\sigma_{n}^{2}=10^{-{\mathrm{SNR}}_{\mathrm{dB}}/10}.$$

Finally, let g(·) denote the classifier at the remote side and $\hat{y}$ its predicted MI label. The receiver operation is

(5) $$\hat{y}=g\left(r\right).$$

The experiment concerns the reliability of this class decision after additive corruption of a compact real-valued vector. It does not simulate quantization, source coding, modulation, channel coding, fading, interference, packet loss, channel estimation, a particular wireless standard, a transmission rate, end-to-end communication latency, or link energy consumption. Accordingly, *K* measures the number of real values transferred per EEG trial, neither a number of bits nor a bit rate. This abstraction deliberately isolates representation robustness: every method is compared under the same vector dimension, power normalization, and additive disturbance, while translation to a deployable digital link remains outside the present model. The next subsection defines how the transmitters construct their messages.

### 2.3. Transmitted EEG Representations

Four approaches construct the vector in Equation (1). The conventional approach summarizes MI activity with established spatial and spectral features. The reconstruction-oriented approach produces a vector designed to retain enough information to recover the original EEG waveform. A supervised filter-bank EEGNet learns a task-oriented message directly from band-limited EEG, whereas the proposed semantic approach learns a bounded refinement of the conventional message. For a given value of *K*, all approaches send exactly *K* real numbers, undergo the same power constraint in Equation (2), and are disturbed according to the same communication model in Equation (3). Their comparison therefore tests how the *content* of an equally sized transmitted vector affects remote classification.

All approaches use the same receiver: a fully connected K→64 layer, an exponential linear unit activation, dropout with probability 0.25, and a four-unit softmax output layer. Holding this architecture constant prevents receiver capacity from being confounded with representation quality.

#### 2.3.1. Conventional FBCSP Representation

The conventional transmitter uses FBCSP, a standard method for MI EEG decoding [9]. The 8–30 Hz range was selected a priori to cover the sensorimotor mu and beta rhythms on which conventional MI decoding is commonly based [5,6]. Frequencies below 8 Hz were not assumed to be universally uninformative; they were excluded to keep all message constructions aligned with this motor-rhythm hypothesis and to reduce sensitivity to slow drift, ocular activity, and cue-locked low-frequency responses. Evaluating broadband and explicitly low-frequency message representations is a separate extension. The analyzed range was divided into five subbands: 8–12, 12–16, 16–20, 20–24, and 24–30 Hz. Within each band, CSP filters were constructed separately for each class against the remaining three classes. Four spatial components were retained for every one-versus-rest (OVR) problem, and the logarithm of the average signal power of each retained component was calculated. The five bands, four class comparisons, and four components yielded 5×4×4=80 features per trial.

Within each OVR problem, CSP used covariance matrices estimated from concatenated trials, Ledoit–Wolf regularization [35], no trace normalization, and mutual-information component ordering. After training-set standardization, PCA using full singular value decomposition reduced the 80 features to the selected value of *K*. Let $u\in R^{K}$ denote this PCA output before communication normalization. The power-normalized conventional vector is

(6) b=PN(u),

where PN(·) denotes Equation (2). The vector b, whose mean squared value is one, forms the conventional message sent through the communication channel.

#### 2.3.2. Exploratory Reconstruction-Oriented Representation

The reconstruction transmitter is a convolutional autoencoder. Its encoder compresses an 8–30 Hz EEG trial into a *K*-dimensional latent vector, while its decoder reconstructs the standardized trial from the noisy latent representation. Reconstruction training therefore encourages the transmitted vector to retain waveform information. After training, the decoder is discarded and the encoded vector is passed to the common receiver. Exact layer dimensions are reported in Table 1. Because this comparator differs from FBCSP–PCA in both input representation and encoder architecture, it contrasts the complete tested reconstruction-oriented pipeline with the task-oriented pipelines; it is not a definitive test of reconstruction learning in general.

To verify that its classification behavior did not arise from corrupted or incorrectly loaded checkpoints, all 162 frozen autoencoder checkpoints (nine subjects, two directions, three seeds, and three values of *K*) were subsequently evaluated without retraining on held-out opposite-session trials in the noise-free condition. Before evaluation, the expected checkpoint count, file size, and SHA-256 checksum of every file were verified against the frozen archive manifest; all 162 checkpoints passed these checks. Reconstruction quality was quantified by normalized mean squared error (NMSE), defined as mean squared error (MSE) divided by the mean squared standardized target, samplewise Pearson correlation, and the relative reduction in MSE compared with predicting the channel-wise mean estimated from the training set.

#### 2.3.3. Supervised Filter-Bank EEGNet Comparator

An additional supervised neural transmitter was evaluated to determine whether an independently learned message could provide a competitive reference under the same communication abstraction. Five band-limited versions of each 22-channel EEG trial, filtered in the same subbands used by FBCSP, were processed by parallel EEGNet branches. Each branch applied temporal convolution, depthwise spatial filtering, a depthwise-separable temporal convolution, and temporal pooling. The five outputs were concatenated and fused before a final projection produced K∈{16,32,64} real values. These values were power-normalized and transmitted through the AWGN channel before classification by the common receiver. The same subjects, bidirectional cross-session split, test SNRs, repetition seeds, and 20 test noise realizations were used as in the main analysis. This comparator is reported as an exploratory end-to-end learned alternative; the proposed contribution remains the baseline-preserving residual design.

#### 2.3.4. Proposed Residual Semantic Representation

The proposed approach begins with the normalized conventional vector b in Equation (6). A residual transformation h(·), comprising layer normalization and two fully connected layers separated by a Gaussian error linear unit, produces a residual vector. With residual scale α, the transmitted semantic vector is

(7) $$s=\mathrm{PN}\left[b+\alpha \tanh\left(h(b)\right)\right].$$

The model used hidden width H=96 and residual scale α=0.35, fixed across subjects, cross-session directions, communication loads, and test conditions. Because the hyperbolic tangent lies between −1 and 1, each pre-normalization correction component is bounded in magnitude by α. No subject-specific or communication-condition-specific selection of these values was performed.

The final layer of h(·) is initialized with zero weights and biases, and the semantic receiver is initialized from the corresponding trained conventional receiver. Before semantic training, Equation (7) therefore yields s=b, and the complete semantic system reproduces the corresponding conventional system. Semantic learning begins from this baseline-preserving state rather than from an unrelated representation and classifier.

#### 2.3.5. Classifier-Only Control

A classifier-only control was evaluated at every K∈{16,32,64} to distinguish modification of the transmitted vector from additional receiver training. Its conventional vector b remained fixed, and only the receiver parameters were updated. In the figures, this condition is labeled Receiver-only.

Table 1 summarizes the learned components; Conv1D denotes a one-dimensional convolution, Conv2D a two-dimensional convolution, and TConv1D a one-dimensional transposed convolution.

These representations were evaluated using the common cross-session protocol described next.

### 2.4. Cross-Session Experimental Design and Input Preparation

#### 2.4.1. Session and Run Partitioning

All analyses were subject-specific: data from different subjects were never combined to fit a representation or classifier. For each subject, the two recording sessions were used in both possible directions. In the first direction, Session 1 supplied the training and validation data, while Session 2 was the held-out test session. In the second direction, their roles were reversed. This produced two cross-session evaluations per subject and ensured that every final prediction concerned a recording day not used to fit or select the corresponding model.

Within the source session, five complete acquisition runs formed the training set used to fit preprocessing transformations and model parameters. The remaining complete run formed the validation set used to select the retained model state. The validation run was chosen deterministically with split seed 2026, with the requirement that all four classes were present in both sets. Keeping runs intact prevented trials from the same recording block from appearing in both training and validation. The opposite session was reserved exclusively as the test set.

The data partition was established before any data-dependent operation was fitted. Within this partition, the signals and method-specific inputs were prepared as follows.

#### 2.4.2. Signal Preprocessing and Method-Specific Inputs

The dataset was accessed with version 1.5.0 of the Mother of All BCI Benchmarks (MOABB) and processed through its MNE-Python-based paradigm interface [33,34]. We specified the filtering, resampling, and epoching parameters; the MOABB/MNE-Python pipeline executed these signal operations and returned the trial arrays together with their session, run, class, and artifact annotations from the source files.

Signals were resampled from 250 to 160 samples/s and analyzed in the pre-specified 8–30 Hz range. Each trial covered the 3.5 s interval 0.5≤t<4.0 s after the MI class cue. Excluding the first 0.5 s reduced the contribution of the immediate visual cue response and retained sustained imagery activity. Consequently, every 8–30 Hz trial used by the reconstruction-oriented approach contained 22 EEG electrodes and 560 temporal samples, or 12,320 signal values. For the FBCSP–PCA, filter-bank EEGNet, and semantic approaches, the same interval was extracted separately in the five frequency bands defined in Section 2.3.1.

Trials carrying an artifact flag in the original BNCI2014-001 source files were identified through an explicit audit that mapped the provider’s flags to the MOABB trial metadata using subject, session, run, within-run trial order, and class label. Flagged trials were excluded before model fitting or evaluation. This removed 488 of the original 5184 trials and retained 4696 trials. No additional automated amplitude-based rejection criterion was introduced, and the same retained trials were used by all methods.

All data-dependent operations were estimated from the training set only. For the reconstruction-oriented and filter-bank EEGNet approaches, each EEG electrode and frequency band was standardized using statistics estimated from the training trials; the same transformations were then applied to the validation and test sets. For the conventional and semantic approaches, the CSP spatial filters, the standardizer for the 80 FBCSP features, and the PCA transformation were fitted only to the training trials and then applied unchanged to validation and test trials. The resulting PCA vectors were power-normalized as specified in Equation (6). Once these leakage-free inputs had been prepared, the learned components were trained while being exposed to the communication disturbances defined earlier.

### 2.5. Model Training and Selection

Learned components expected to operate with noise-corrupted vectors were exposed to such disturbances during training. Accordingly, the AWGN operation in Equation (3) remained active during training. For each training example, an SNR was sampled from S={−10,−5,0,5,10,15,20} dB.

This procedure allowed one model to operate across several noise levels instead of fitting a separate model for each SNR. Three deterministic repetitions used seeds 2026, 2027, and 2028. For the semantic and classifier-only models, a distinct initialization seed was deterministically derived for every subject, cross-session direction, communication load, and repetition seed before model construction. Training-batch order and the two semantic training stages were also controlled by deterministic seeds.

Classification models were learned using categorical cross-entropy, which measures disagreement between the predicted class probabilities and the known MI label. The criterion used to assess predictions on the validation set was balanced accuracy. For a class *c*, let ${\mathrm{TP}}_{c}$ denote its correctly recognized trials and ${\mathrm{FN}}_{c}$ its missed trials. Balanced accuracy was defined as the mean class-specific recognition rate:

(8) $$\mathrm{BA}=\frac{1}{4}\sum_{c=1}^{4}\frac{{\mathrm{TP}}_{c}}{{\mathrm{TP}}_{c}+{\mathrm{FN}}_{c}}.$$

Balanced accuracy therefore gives the same weight to every MI class.

For the conventional approach, the common receiver classifier was trained from noisy FBCSP–PCA vectors. The SNR of each example was sampled uniformly from S. At the end of every epoch, balanced accuracy was calculated on the validation set at all seven noisy SNRs, using one fixed disturbance, the same generated noise values at every epoch, for each SNR. The retained parameter state was the one with the highest mean balanced accuracy across these seven conditions.

For the reconstruction-oriented approach, the encoder and decoder were first trained together. The power-normalized reconstruction-oriented vector was corrupted at an SNR sampled uniformly from S, and the decoder attempted to recover the standardized EEG trial. The training objective was mean squared error, calculated as the average squared difference between corresponding samples of the original and reconstructed trials. Model selection used the mean reconstruction error across the seven noisy SNRs on the validation set. The selected encoder was then fixed, the decoder was removed, and the common receiver classifier was trained and selected from the noisy reconstruction-oriented vectors in the same manner as the conventional receiver.

The filter-bank EEGNet transmitter was first pretrained on clean trials using cross-entropy together with a supervised contrastive term of weight 0.05. Robust joint fine-tuning then optimized the transmitter and common receiver using clean-trial cross-entropy with weight 0.25, the mean cross-entropy of two independently noise-corrupted views with weight 0.75, and the same contrastive weight of 0.05. The contrastive temperature was 0.1, classification targets used label smoothing 0.05, and noisy SNRs were sampled from S. Model selection combined noise-free validation performance with weight 0.25 and mean robust validation performance with weight 0.75. Clean pretraining used Adam at $10^{-3}$; robust fine-tuning used rates of $3\times 10^{-4}$ for the encoder and $5\times 10^{-4}$ for the receiver. Early-stopping patience was 30 epochs for pretraining and 35 for robust fine-tuning.

The semantic transmitter was optimized to retain correct decisions across several noise conditions while remaining close to its conventional starting point. Five quantities were defined for this purpose. First, $L_{\mathrm{clean}}$ was the cross-entropy obtained from the semantic vector without added noise. Second, $L_{\mathrm{severe}}$ was the mean cross-entropy from two independently disturbed copies of each vector, where the SNR of each copy was sampled from {−10,−5} dB. Third, $L_{\mathrm{mixed}}$ was the cross-entropy from one additional disturbed copy whose SNR was sampled from the complete set S. Fourth, the anchor term was

(9) $$L_{\mathrm{anchor}}=\frac{1}{K}{∥s-b∥}_{2}^{2}.$$

It penalized unnecessary departure of the semantic vector s from the normalized conventional vector b. Fifth, $L_{\mathrm{distill}}$ encouraged the semantic system to preserve the class-probability structure produced by a fixed copy of the conventional system. To obtain this term, the output scores of both systems were divided by a temperature τ=2 before conversion to probabilities. This operation produces flatter probability distributions and exposes similarities among the non-selected classes. Let $p_{\mathrm{conv},c}^{\left(\tau \right)}$ and $p_{\mathrm{sem},c}^{\left(\tau \right)}$ denote the resulting conventional and semantic probabilities for class *c*. The probability-preservation term was the temperature-scaled Kullback–Leibler divergence [36]:

(10) $$L_{\mathrm{distill}}=\tau^{2}\sum_{c=1}^{4}p_{\mathrm{conv},c}^{\left(\tau \right)}\log\frac{p_{\mathrm{conv},c}^{\left(\tau \right)}}{p_{\mathrm{sem},c}^{\left(\tau \right)}}.$$

Having defined all five quantities, the semantic training objective was

(11) $$\begin{array}{cc} L_{\mathrm{semantic}}= & 0.75L_{\mathrm{clean}}+0.75L_{\mathrm{severe}}+0.50L_{\mathrm{mixed}}\\ \; & \;+0.35L_{\mathrm{anchor}}+0.50L_{\mathrm{distill}}. \end{array}$$

The weights prioritized clean and severe-noise classification, retained exposure to the complete noisy SNR range, and constrained departure from the conventional representation and its class-probability structure. They were selected during exploratory method development and then held fixed throughout the reported all-nine-subject execution; they were not adapted separately by subject, direction, communication load, or test condition. This weighting makes severe-noise robustness an explicit design target rather than a claim of uniform improvement over all SNRs.

For each of the three classification terms, the target that would ordinarily assign all probability to the correct class was replaced by a slightly smoothed target distribution with smoothing factor 0.02. This label-smoothing operation reduced absolute confidence in a single class during optimization. Training then proceeded in two stages. The receiver was fixed during the first stage, so only the residual transformation learned to modify the FBCSP–PCA vector; this stage is termedresidual warm-up. During the second stage, termed joint refinement, the residual transformation and receiver were refined together, using a smaller learning rate for the receiver.

At each epoch, semantic validation used three fixed noise realizations at every SNR in S and a noise-free condition in which n=0. Let ${\mathrm{BA}}_{q}$ denote validation balanced accuracy at SNR *q*, and let ${\mathrm{BA}}_{\mathrm{NF}}$ denote validation balanced accuracy without communication noise. The semantic model-selection score *U* emphasized the two most difficult conditions:

(12) $$U=\frac{{\mathrm{BA}}_{-10}+{\mathrm{BA}}_{-5}}{2}+0.1\frac{{\mathrm{BA}}_{0}+{\mathrm{BA}}_{\mathrm{NF}}}{2}.$$

A parameter state was eligible for selection only if its balanced accuracies at 0 dB and in the noise-free condition were no more than 0.01 (one percentage point) below those of the corresponding conventional model. Because the semantic system was initialized to reproduce the conventional system, an unchanged conventional state was eligible from the start; a semantic refinement was retained only when it satisfied this 0 dB/noise-free reference-condition guard. The guard is therefore a design constraint, and validation-set proximity to the conventional model in those two reference conditions is not treated as an independently discovered performance benefit.

The classifier-only control used the same clean, severe-noise, mixed-noise, and probability-preservation terms but omitted $L_{\mathrm{anchor}}$, because its transmitted vector remained fixed. It was selected using the same score and reference-condition guard as the semantic model.

Table 2 summarizes the optimization settings. During semantic and classifier-only training, any gradient vector with a norm above 5 was rescaled to have a norm of 5, preventing an unusually large parameter update from destabilizing optimization.

For the Adam and AdamW stages, weight decay was $10^{-4}$ and batch size was 32. An improvement smaller than $10^{-4}$ was ignored. Early-stopping patience was 30 epochs unless otherwise stated; it was 35 for EEGNet robust fine-tuning and 40 for semantic joint refinement and the classifier-only control. The learning rate was halved after 10 epochs without improvement, down to $10^{-5}$. Only the parameter state selected from the source session was subsequently applied to the held-out test session.

### 2.6. Robustness and Computational Analyses

Six sensitivity variants were evaluated at K=32 with all nine subjects while keeping the architecture, data partitions, seeds, and test procedure fixed. The severe-noise loss weight was multiplied by 0.5 or 1.5; the anchor and distillation weights were jointly multiplied by 0.5 or 1.5; the 0 dB/noise-free reference-condition guard was removed; and one variant replaced severe-SNR sampling and low-SNR selection with uniform sampling and mean validation balanced accuracy across all seven noisy SNRs. These variants test whether the reported effect depends on a single loss weighting, the low-SNR checkpoint score, or the reference-condition guard. They were sensitivity checks and were not used to replace the frozen primary model.

The final residual configuration was selected in a late-stage pilot gate using Subjects 1–3 and then frozen before the full Subjects 1–9 execution. To assess sensitivity to this development sequence without redefining the primary cohort, the mean semantic-minus-conventional gain over the nine low-SNR budget–SNR combinations was summarized separately for Subjects 1–3 and for Subjects 4–9, which were not used in that gate. The same gain was also recomputed after omitting each subject in turn. These are internal sensitivity analyses, not independent-dataset validation.

Architecture-level computational cost was measured on one central processing unit (CPU) thread of an Intel Xeon processor at 2.20 GHz, with a batch size of one. After 100 warm-up passes, 1000 forward passes were timed for every neural path. We report trainable parameters, multiply–accumulate operations (MACs) per trial, and median forward latency with 5–95th percentiles. FBCSP–PCA and filter-bank signal preprocessing were excluded from this benchmark, so the values compare learned message and receiver blocks rather than end-to-end device latency or energy consumption.

### 2.7. Evaluation and Statistical Analysis

Each selected model was evaluated on the held-out test session at every noisy SNR in S, together with a noise-free condition. At every noisy SNR, 20 independently generated disturbances were applied to each test trial. The same deterministic disturbances were used for corresponding methods so that paired methods were compared under identical communication conditions. The noise-free condition was evaluated once per test trial.

Balanced accuracy from Equation (8) was the classification outcome, whereas *K* quantified the communication load. At each noisy SNR, balanced accuracy was first averaged across the 20 noise realizations. The noise-free score was used directly. For every subject, method, value of *K*, and test condition, results were then averaged across the three deterministic repetitions and the two cross-session directions. This produced one value per subject for every experimental condition. Subjects, rather than individual trials, directions, repetitions, or noise realizations, were treated as the independent units of group analysis.

Group values are reported as means across the nine subjects. For descriptive performance curves, conventional two-sided *t*-based 95% confidence intervals were calculated across subjects without multiplicity adjustment; inferential conclusions relied exclusively on paired method differences and the max-|t|-adjusted *p*-values defined below. Method comparisons were performed on paired subject-level differences. Because the independent sample contained nine subjects and the 24 conditions within a comparison family were strongly dependent, primary inference used an exact joint subject-wise sign-flip max-|t| procedure [37]. The sign-flip procedure assumes independent subjects and joint sign-symmetry of their paired-difference vectors under the null. For each comparison family, the sign of every subject’s complete vector of paired differences was either retained or reversed, and all $2^{9}=512$ joint sign patterns were enumerated. Applying one sign to all conditions from the same subject preserved their cross-condition dependence. For each sign pattern, the largest absolute paired *t* statistic across the family was retained. The max-|t|-adjusted *p*-value for a condition was the proportion of the 512 maxima at least as large as its observed absolute *t* statistic. For each paired mean difference, a descriptive two-sided 95% confidence interval was calculated as the mean difference plus or minus $t_{0.975,8}$ times its subject-level standard error, where $t_{0.975,8}$ is the 97.5th percentile of the *t* distribution with eight degrees of freedom. These pointwise intervals assume approximately normal subject-level differences, are not multiplicity-adjusted, and were not used to determine significance. Under the stated sign-symmetry assumption, the exact max-|t| tests control the family-wise error rate over every condition in the family.

The primary family compared the semantic and conventional FBCSP–PCA approaches across three values of *K* and eight channel conditions (24 tests). Corresponding 24-condition families compared the semantic approach with the reconstruction-oriented and receiver-only comparators and the conventional approach with those comparators. An additional exploratory 24-condition family compared conventional FBCSP–PCA with the supervised filter-bank EEGNet transmitter. A two-sided family-wise threshold of 0.05 was used. Paired *t*-tests with Holm correction and marginal exact sign-flip tests with Holm correction were retained as statistical-method sensitivity analyses and are reported in Appendix A.

## 3. Results

The final analysis contained every defined condition for each method at K∈{16,32,64}, and no subject-level result was missing. For concise reporting, the −10, −5, and 0 dB conditions are referred to as the low-SNR region.

### 3.1. Classification Performance Across Channel Conditions and Transmission Budgets

**Figure 2:**
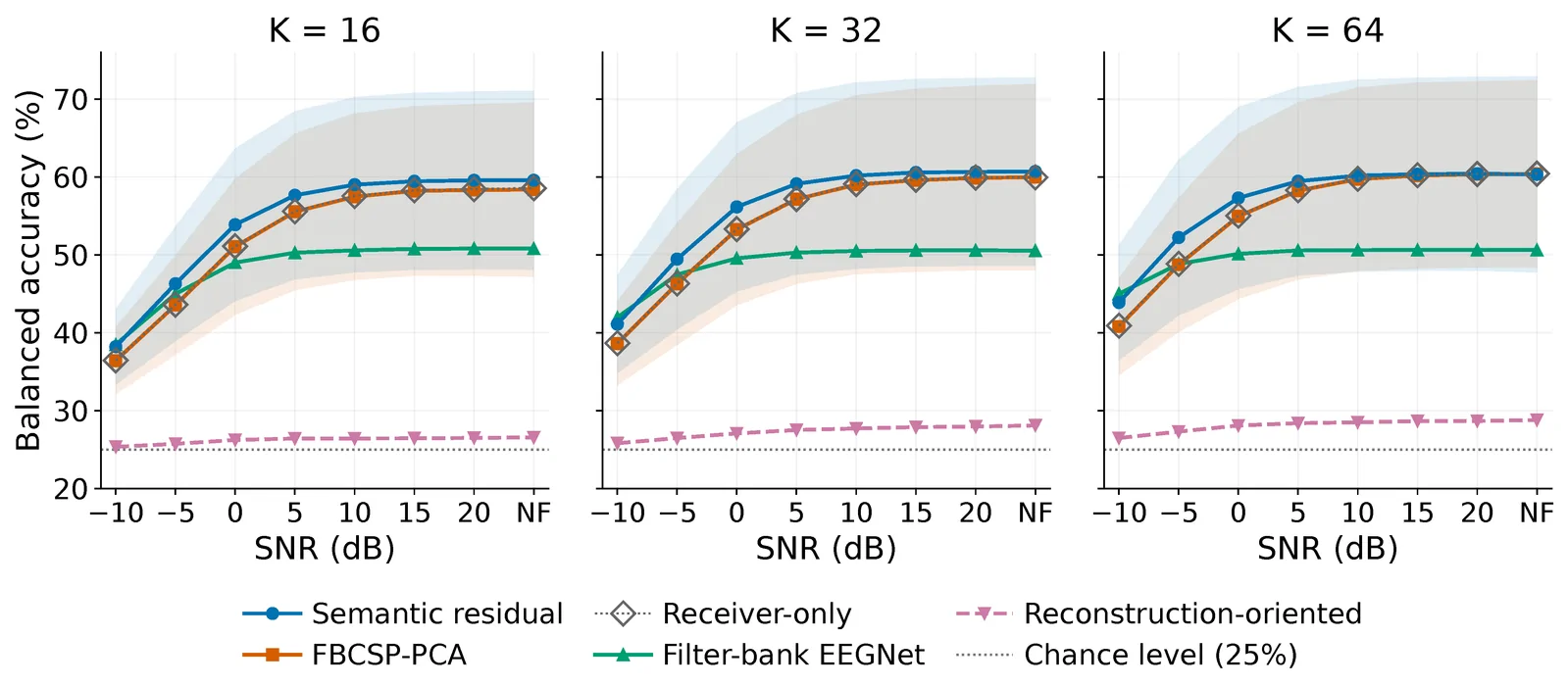
Balanced accuracy across message dimensions for all five comparison methods. Bands show descriptive 95% confidence intervals for the semantic and FBCSP–PCA means; NF, noise-free.

Figure 2 presents the group results across all three transmission budgets; the middle panel corresponds to K=32. At −10, −5, and 0 dB, the proposed semantic approach obtained mean balanced accuracies of 41.10%, 49.46%, and 56.15%, respectively, compared with 38.63%, 46.28%, and 53.26% for conventional FBCSP–PCA. The receiver-only control obtained 38.66%, 46.32%, and 53.31%, while the filter-bank EEGNet obtained 41.97%, 47.47%, and 49.56%. The corresponding reconstruction-oriented values were 25.81%, 26.47%, and 27.07%. At 20 dB, the semantic and conventional group means were 60.65% and 59.87%, respectively; in the noise-free condition, they were 60.71% and 59.98%.

At −5 dB, semantic balanced accuracy increased from 46.30% at K=16 to 52.22% at K=64, while conventional accuracy increased from 43.55% to 48.73%. The receiver-only curves remained visually aligned with FBCSP–PCA at every budget. Across the nine low-SNR budget–SNR combinations, the filter-bank EEGNet mean was 46.16%, compared with 45.96% for FBCSP–PCA and 48.73% for the semantic residual. In the noise-free condition, semantic and conventional performance ranged from 59.58% to 60.71% and from 58.38% to 60.39%, respectively.

### 3.2. Primary Comparison with Conventional FBCSP–PCA Transmission

**Figure 3:**
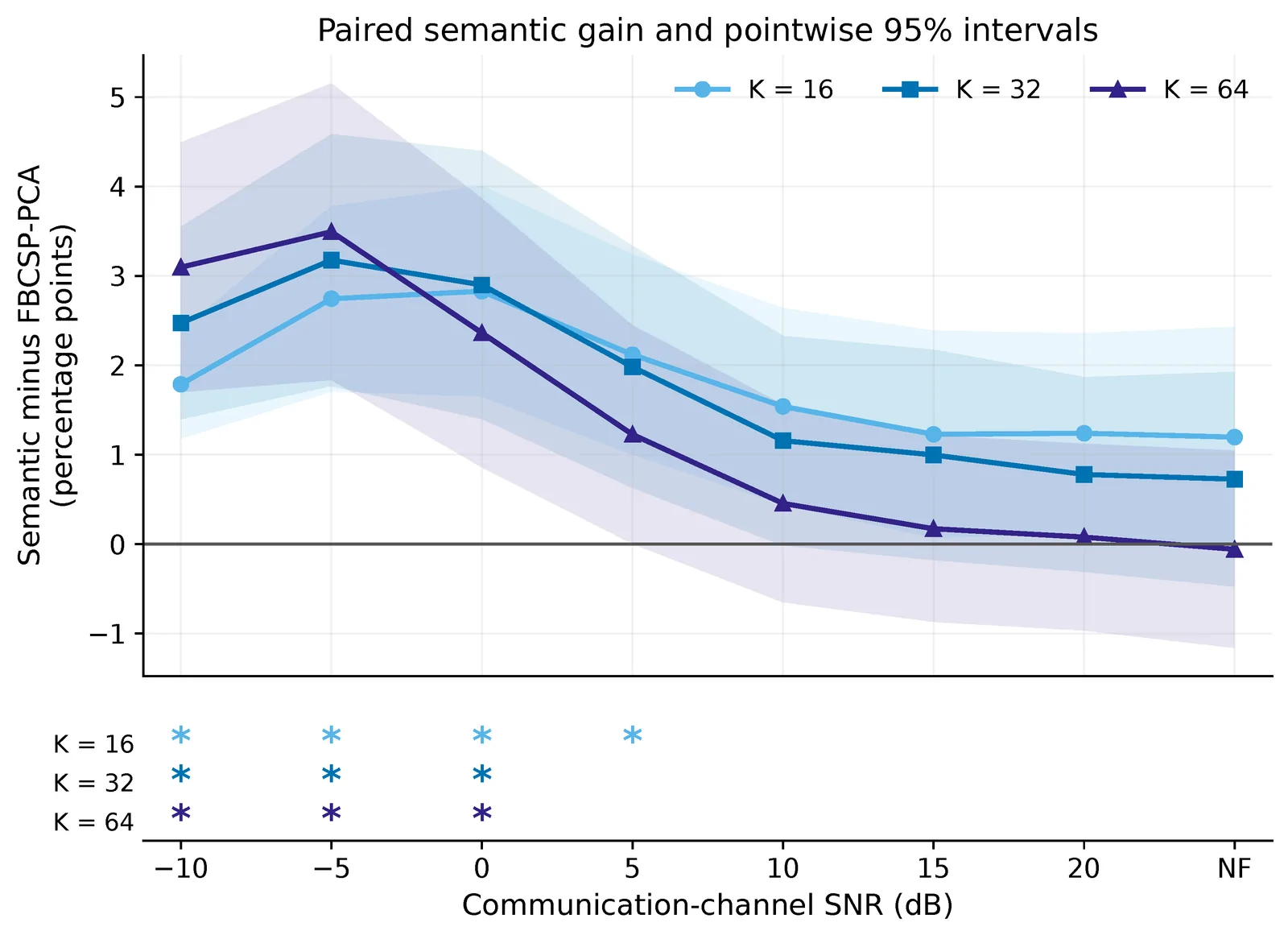
Semantic-minus-FBCSP–PCA balanced-accuracy difference. Bands show descriptive pointwise 95% confidence intervals; asterisks in the lower rows denote exact max-|t|-adjusted p<0.05; NF, noise-free.

The mean semantic-minus-conventional difference was positive in all nine low-SNR budget–SNR combinations (Figure 3; Table 3). All nine subjects favored the semantic approach in eight combinations; at K=64 and 0 dB, eight of nine favored it.

Exact joint max-|t| inference identified 10 significant conditions among the 24 semantic-versus-conventional comparisons. All nine low-SNR combinations were significant, and the remaining significant condition was K=16 at 5 dB, with a 2.12 percentage-point mean difference (descriptive 95% confidence interval, 1.00 to 3.24; $p_{\max T}=0.0156$). No comparison at 10, 15, or 20 dB or in the noise-free condition was significant. In the noise-free condition, the mean semantic-minus-conventional differences were 1.20, 0.73, and −0.06 percentage points for K=16, K=32, and K=64, respectively.

### 3.3. Reconstruction, Learned-Transmitter, and  Receiver-Only Comparisons

The reconstruction-oriented classifier produced balanced accuracies between 25.35% and 28.79% across the reported conditions. The frozen-checkpoint diagnostic nevertheless verified that its encoder–decoder path retained modest waveform information on the held-out opposite session. Across K=16,32,64, mean NMSE was 0.961, 0.960, and 0.959; mean Pearson correlation was 0.171, 0.175, and 0.189; and mean MSE reduction relative to the training-set channel-mean predictor was 3.87%, 4.01%, and 4.06%, respectively. The corresponding 95% intervals for MSE reduction were [0.83, 6.91]%, [0.98, 7.04]%, and [1.32, 6.80]%, respectively; eight of nine subjects improved on the predictor at every *K*. Thus, the archived checkpoints were functional, although the retained waveform structure supported weak MI classification in this implementation.

The supervised filter-bank EEGNet produced a mean low-SNR difference of 0.20 percentage points relative to FBCSP–PCA when the nine budget–SNR combinations were pooled. Four of nine subjects had a positive pooled difference (exact sign-flip p=0.9180). Its relative performance was highest at −10 dB but declined as SNR increased; under an exploratory exact 24-condition max-|t| family, no positive low-SNR comparison was significant, while FBCSP–PCA was significantly higher in 13 moderate-to-high-SNR conditions. This learned transmitter therefore provided an informative comparison without uniformly reproducing the residual method’s behavior.

Receiver-only training was extended to all three message dimensions. Across its 24 budget–condition combinations the absolute group-mean difference from conventional FBCSP–PCA never exceeded 0.174 percentage points. Two K=16 differences were statistically detectable because their between-subject variance was very small, but their magnitudes were only 0.046 and 0.102 points. By contrast, the semantic approach retained positive low-SNR differences relative to receiver-only at every *K*, and exact max-|t| inference identified all nine low-SNR comparisons plus K=16 at 5 dB as significant. Table 4 reports the low-SNR conditions.

### 3.4. Sensitivity and Computational Results

At K=32, the primary model’s mean semantic-minus-conventional gain averaged over −10, −5, and 0 dB was 2.85 percentage points. The corresponding gains were 2.42 points with uniform all-SNR training and checkpoint selection, 2.56 and 2.93 points after multiplying the severe-noise weight by 0.5 and 1.5, 2.88 and 2.87 points after multiplying the anchor and distillation weights by 0.5 and 1.5, and 3.43 points after removing the 0 dB/noise-free reference-condition guard. Every variant yielded positive low-SNR gains for all nine subjects (exact sign-flip p=0.0039); the no-guard variant retained a positive noise-free difference of 0.86 points. The low-SNR gain therefore did not depend on one weight, the original checkpoint utility, or the reference-condition guard.

Across all nine subjects, the mean gain over the nine low-SNR budget–SNR combinations was 2.76 percentage points; all nine subjects had positive average gains (exact p=0.0039). The corresponding gain was 3.54 points for Subjects 1–3 (3/3 positive; exact p=0.2500) and 2.37 points for Subjects 4–9 (6/6 positive; exact p=0.0313). Leave-one-subject-out (LOSO) gains ranged from 2.50 to 3.00 points, and every eight-subject analysis remained positive (exact p=0.0078).

At K=32, the common receiver contained 2372 trainable parameters and required 2304 MACs per trial, whereas the semantic residual-plus-receiver path contained 8708 parameters and required 8448 MACs. Their median single-thread CPU forward latencies were 0.038 and 0.158 ms, respectively. The reconstruction classification path required 7.56 million MACs and 1.276 ms, while the filter-bank EEGNet classification path required 32.55 million MACs and 7.809 ms. These values exclude FBCSP–PCA and filter-bank preprocessing and therefore describe model-block complexity rather than total acquisition-to-decision cost.

### 3.5. Subject-Level Consistency

**Figure 4:**
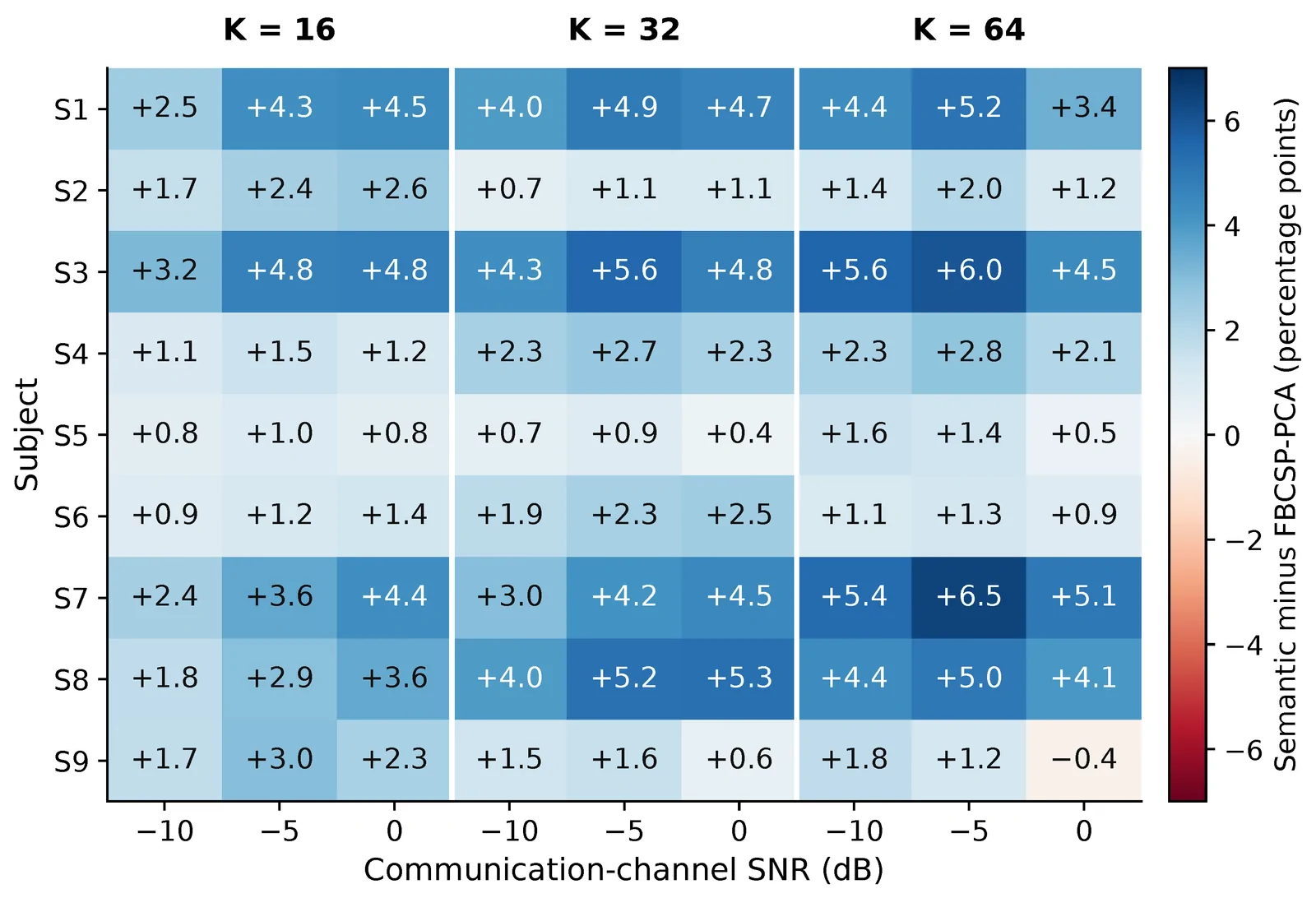
Subject-level semantic-minus-FBCSP–PCA balanced-accuracy differences at low SNR (percentage points).

Figure 4 shows the paired differences for every subject and low-SNR condition. Of the 81 subject–budget–SNR cells, 80 favored the semantic approach; the only negative difference was −0.37 percentage points for Subject 9 at K=64 and 0 dB.

## 4. Discussion

### 4.1. Principal Findings

Under matched message dimension, power normalization, held-out test trials, and AWGN realizations, task-oriented residual refinement improved balanced accuracy primarily at low SNR. Exact family-wise inference identified all nine low-SNR comparisons and one 5 dB comparison as significant, with low-SNR gains of 1.79–3.49 percentage points. Receiver-only controls closely reproduced conventional performance in magnitude at every message dimension, while the low-SNR effect remained positive under uniform-SNR training and selection, altered loss weights, removal of the reference-condition guard, and LOSO analyses. These convergent controls associate the observed gain with refinement of the transmitted representation rather than classifier retraining alone or one particular hyperparameter setting.

The study was designed to compare message constructions, not to propose a new state-of-the-art MI classifier. More elaborate EEG decoders can achieve higher absolute accuracy on the BNCI2014-001 dataset, but changing classification capacity would obscure the controlled communication comparison. The deliberately compact residual design starts from the same conventional message and receiver, enabling the effect of modifying the transmitted representation to be isolated. Its architectural simplicity is therefore part of the experimental control and supports reproducible transfer of the protocol to other datasets and paradigms. The CPU analysis quantifies forward-path complexity but was not intended as a hardware-optimization benchmark. A recent perspective on MI-based BCIs likewise emphasizes reconsidering task-relevant neurophysiological features rather than focusing only on increasingly sophisticated decoding algorithms [38].

### 4.2. Interpretation of the Low-SNR Benefit

The semantic objective prioritized the downstream MI decision over faithful recovery of the original EEG waveform, following the task-oriented view that a message is valued by its usefulness for the receiver task rather than solely by sample- or symbol-level fidelity [22,23,24]. The present method applies that principle conservatively by beginning with FBCSP–PCA, an established spatial–spectral representation for MI decoding, and learning only a bounded correction [5,9].

After FBCSP and PCA, the conventional mapping remains fixed; noise-aware receiver training can alter only the decision boundary. Semantic training can also modify the representation while observing noisy instances. Its emphasis on −10 and −5 dB promotes robustness under severe disturbance, whereas the anchor and distillation terms limit departure from the conventional solution.

The concentration of benefit at low SNR is consistent with this declared optimization target and should not be interpreted as uniform dominance. Importantly, the uniform-SNR variant retained a positive mean gain across the three low-SNR tests, while removal of the 0 dB/noise-free reference-condition guard increased rather than eliminated that gain and preserved a positive noise-free difference. The observed effect therefore cannot be attributed solely to the original checkpoint score or to mechanically enforcing proximity at the two guarded conditions. Proximity on the validation set was imposed by the stated design constraint rather than established independently.

A plausible explanation is that the residual transformation rearranged the compact FBCSP–PCA vectors so that task-relevant class distinctions were less vulnerable to the additive perturbations encountered during training. Such a rearrangement may increase class margins at vulnerable decision boundaries or distribute class-relevant evidence more robustly across the *K* transmitted components. This interpretation is consistent with the paired low-SNR gains and with task-oriented communication studies in other data modalities, where representations optimized for the receiver task retained greater utility under restrictive or noisy links [21,23,24].

Existing neural semantic-communication studies address different receiver tasks, including multi-brain robot control, visual-perception decoding, multi-task EEG representation, and adversarially robust brain-to-brain communication [27,28,29,30]. Direct accuracy comparisons would therefore be uninformative because their datasets, labels, and links differ. The present contribution is complementary: it isolates baseline-preserving refinement of an established MI representation under a fixed message size and matched additive disturbances.

The reduction of the difference at higher SNRs is equally informative. When little or no communication noise was added, the conventional representation was already near its performance plateau, leaving less scope for channel-aware refinement. Moreover, zero initialization of the residual path, the anchor loss, the distillation loss, and the reference-condition guard were designed to retain the conventional solution when a change was unnecessary.

### 4.3. Evidence from the Comparison Methods

The receiver-only control isolates receiver adaptation from message refinement. The negligible receiver-only changes, together with the semantic method’s significant low-SNR advantage over this control at every *K*, indicate that additional receiver optimization alone did not account for the improvement.

The reconstruction comparison addresses a different objective. The held-out diagnostic confirmed that the frozen autoencoders retained modest waveform structure and improved MSE relative to the training-set channel-mean predictor, ruling out checkpoint corruption or a nonfunctional decoder as the explanation for their classification results. Nevertheless, their waveform recovery was weak, and the reconstruction system differed from FBCSP–PCA in input representation and architecture. Its near-chance MI classification therefore characterizes this exploratory implementation under a severe *K*-dimensional bottleneck; it does not establish that reconstruction learning is generally unsuitable for EEG. Suitably designed autoencoder features can support MI decoding [15], and stronger reconstruction or hybrid task–reconstruction objectives remain relevant comparators.

The supervised filter-bank EEGNet supplies a directly learned task-oriented message at the same values of *K*. Its small pooled low-SNR difference from FBCSP–PCA and lower moderate-to-high-SNR performance show that end-to-end supervision alone did not reproduce the residual approach’s consistent behavior. This does not establish a general ranking of neural and handcrafted EEG representations; rather, it highlights the value of preserving a strong conventional starting point in the present small-sample cross-session setting.

Increasing *K* generally raised balanced accuracy for both the conventional and semantic approaches at low SNR, but the semantic-minus-conventional difference did not increase monotonically with *K*. These are separate effects: sending more real values supplied a larger representation to both methods, whereas task-oriented refinement altered the content of a representation at a fixed size. The within-budget comparisons therefore show that the semantic gains were not obtained by transmitting a longer vector.

### 4.4. Across-Subject Consistency

Positive semantic-minus-conventional differences in 80 of the 81 low-SNR subject–budget–SNR cells show that the group effect was broadly distributed rather than driven by a small number of individuals. The Subjects 4–9 and LOSO analyses further show that this direction did not depend on the late-stage pilot subset or one influential participant. These absolute gains are modest and do not by themselves establish clinical or operational benefit. Their value depends on application-specific factors such as error cost, decision cadence, user adaptation, and link conditions; here they demonstrate that representation refinement recovered a reproducible portion of decision performance under severe additive corruption at unchanged message dimension.

### 4.5. Study Scope and Future Directions

The findings apply to subject-specific cross-session analysis of one nine-subject, four-class MI benchmark under an analog AWGN abstraction. This controlled link isolates additive representation corruption, but it does not implement a complete wireless system. Equal *K* means equal real-valued vector dimension, not equal quantized bit rate, latency, bandwidth, or energy. Quantization can discard small residual adjustments; modulation and channel coding can redistribute protection across message components; fading and interference introduce multiplicative or structured disturbances; and packet loss, channel estimation, and retransmission create effects absent from Equation (3). The reported SNRs therefore should not be translated directly into deployment link margins. Their role is to provide a common disturbance scale for comparing message constructions.

All nine subjects in the BNCI2014-001 dataset were included, but nine independent units still limit population precision. Exact subject-wise inference avoids a low-powered normality decision, and the subject-level sensitivity analyses show that the low-SNR direction is not driven by one influential participant; they do not replace validation on an independent cohort. Participant characteristics such as handedness and hemispheric dominance were unavailable in the public release. Method development and final evaluation also used the same benchmark, so the present evidence is internal and cross-session rather than external.

The architecture benchmark shows that the residual message path is lightweight relative to the two learned encoders, but it excludes signal preprocessing and does not measure device energy. Future work should apply the frozen procedure to an independent MI dataset, incorporate quantization and representative fading or interference models, and measure end-to-end latency and energy on acquisition hardware. Broadband or low-frequency messages, conventionally compressed raw EEG, and stronger supervised, reconstruction, or hybrid transmitters would further test which task-relevant structures remain most robust under practical communication constraints. The same message-level framework could also be extended to other physiological sensors, but each modality would require its own task, acquisition protocol, and validation rather than being treated as an interchangeable substitute for MI EEG.

## 5. Conclusions

Within the evaluated subject-specific cross-session AWGN setting, baseline-preserving task-oriented refinement of FBCSP–PCA features improved MI decoding primarily at low SNR without increasing the transmitted dimension. Exact family-wise inference supported all nine comparisons at −10, −5, and 0 dB, with gains of 1.79–3.49 percentage points, while all-budget receiver-only controls and loss/model-selection sensitivity analyses associated the effect with message refinement rather than classifier retraining or one particular design constraint. The tested reconstruction pipeline retained modest waveform information but provided weak MI classification, and the independent filter-bank EEGNet transmitter did not uniformly match the baseline-preserving system. These results support lightweight task-oriented refinement as a reproducible strategy for protecting remote EEG decisions under controlled additive noise, while independent datasets and digital, fading, and hardware-level evaluations remain necessary before deployment conclusions are drawn. 

## Figures and Tables

**Table 1:** Neural architectures used in the compared methods.

Component	Architecture
Common receiver classifier	Fully connected K→64; exponential linear unit; dropout 0.25; fully connected 64→4.
Reconstruction encoder	Conv1D 22→32, kernel 15, stride 2, padding 7; Conv1D 32→64, kernel 9, stride 2, padding 4; Conv1D 64→64, kernel 7, stride 2, padding 3; batch normalization, exponential linear unit, and dropout 0.25 after every convolution; adaptive average pooling to eight positions; fully connected 512→K.
Reconstruction decoder	Fully connected K→4480; exponential linear unit; TConv1D 64→64, kernel 7, stride 2, padding 3, output padding 1; TConv1D 64→32, kernel 9, stride 2, padding 4, output padding 1; TConv1D 32→22, kernel 15, stride 2, padding 7, output padding 1; batch normalization and exponential linear unit after the first two transposed convolutions.
Filter-bank EEGNet transmitter	Five parallel branches, each comprising Conv2D 1→8, kernel 1×63; depthwise spatial Conv2D 8→16, kernel 22×1, groups 8; average pooling 1×4; depthwise-separable temporal convolution, kernel 1×15; average pooling 1×8; adaptive pooling to eight temporal positions; dropout 0.35. Branch outputs are concatenated, layer-normalized, and projected through fully connected layers 640→256→K. Trainable parameters, including the common receiver: 177,996, 183,132, and 193,404 for K=16,32,64, respectively.
Semantic residual transformation	Layer normalization; fully connected K→H; Gaussian error linear unit; fully connected H→K, with H=96; residual scale α=0.35 before final power normalization.

**Table 2:** Optimization and model-selection settings.

Stage	Optimizer	Learning Rate	Maximum Epochs	Model-Selection Criterion
Conventional receiver	Adam	$10^{-3}$	300	Mean validation balanced accuracy over the seven noisy SNRs.
Reconstruction autoencoder	Adam	$10^{-3}$	300	Mean validation reconstruction error over the seven noisy SNRs.
Reconstruction receiver	Adam	$10^{-3}$	300	Mean validation balanced accuracy over the seven noisy SNRs.
Filter-bank EEGNet transmitter and receiver	Adam	$10^{-3}$; fine-tuning $3\times 10^{-4}$ encoder and $5\times 10^{-4}$ receiver	300	Weighted clean and robust validation balanced accuracy.
Semantic residual warm-up	AdamW	$5\times 10^{-4}$	160	Equation (12), subject to the reference-condition guard.
Semantic joint refinement	AdamW	$2\times 10^{-4}$ residual; $5\times 10^{-5}$ receiver	220	Equation (12), subject to the reference-condition guard.
Classifier-only control	AdamW	$5\times 10^{-5}$	220	Equation (12), subject to the reference-condition guard.

**Table 3:** Low-SNR comparison of semantic and conventional FBCSP–PCA transmission.

*K*	SNR	Semantic BA (%)	FBCSP–PCA BA (%)	Difference (pp) Pointwise 95% CI	Subjects Favoring Semantic	$p_{\max T}$
16	−10 dB	38.21	36.42	1.79 [1.18, 2.40]	9/9	0.0039
16	−5 dB	46.30	43.55	2.74 [1.71, 3.78]	9/9	0.0039
16	0 dB	53.88	51.05	2.83 [1.65, 4.01]	9/9	0.0039
32	−10 dB	41.10	38.63	2.47 [1.40, 3.55]	9/9	0.0039
32	−5 dB	49.46	46.28	3.18 [1.77, 4.59]	9/9	0.0039
32	0 dB	56.15	53.26	2.90 [1.40, 4.40]	9/9	0.0156
64	−10 dB	43.89	40.79	3.10 [1.70, 4.49]	9/9	0.0039
64	−5 dB	52.22	48.73	3.49 [1.83, 5.15]	9/9	0.0078
64	0 dB	57.31	54.95	2.36 [0.86, 3.87]	8/9	0.0391

Note: BA, balanced accuracy; pp, percentage points; CI, confidence interval. CIs are descriptive pointwise paired *t*-based intervals, without multiplicity adjustment. Only $p_{\max T}$ values determine significance, using the exact joint subject-wise max-|t| procedure across all 24 conditions; bold values are below 0.05.

**Table 4:** Low-SNR comparison with the receiver-only control.

*K*	SNR	Difference (pp) Pointwise 95% CI	Subjects with Positive Difference	$p_{\max T}$
16	−10 dB	1.76 [1.16, 2.36]	9/9	0.0039
16	−5 dB	2.70 [1.66, 3.73]	9/9	0.0039
16	0 dB	2.77 [1.63, 3.91]	9/9	0.0039
32	−10 dB	2.44 [1.37, 3.51]	9/9	0.0039
32	−5 dB	3.14 [1.75, 4.53]	9/9	0.0039
32	0 dB	2.84 [1.35, 4.33]	9/9	0.0078
64	−10 dB	2.98 [1.64, 4.33]	9/9	0.0039
64	−5 dB	3.38 [1.81, 4.95]	9/9	0.0039
64	0 dB	2.28 [0.82, 3.74]	8/9	0.0352

Note: Differences are semantic minus receiver-only in percentage points; CI, confidence interval. CIs are descriptive pointwise paired *t*-based intervals, without multiplicity adjustment. Only $p_{\max T}$ values determine significance, using one exact 24-condition max-|t| family; bold values are below 0.05.

## Data Availability

BNCI2014-001 (BCI Competition IV Dataset 2a) is publicly available from the BNCI Horizon 2020 repository (https://bnci-horizon-2020.eu/database/data-sets, accessed on 8 September 2026) and can be downloaded programmatically through MOABB under the identifier BNCI2014_001. The analysis code is publicly available at https://github.com/HosseinAhmadi63/Semantic-EEG-Communication (accessed on 8 September 2026).

## Appendix A. Additional Robustness and Sensitivity Analyses

The sensitivity variants below were evaluated after the primary configuration had been frozen and were not used to select a replacement model. Paired *t*-tests with Holm correction retained nine primary conditions, whereas marginal exact sign-flip tests followed by Holm correction retained none. The joint max-|t| procedure was designated as primary because it uses exact subject-wise sign flips while preserving dependence across the full 24-condition family; the coarser marginal sign-flip result illustrates the limited attainable resolution when 24 separately adjusted tests are based on nine subjects.

**Table A1:** Sensitivity of the K=32 semantic gain.

Configuration	Low-SNR Gain Mean ± SD (pp)	Positive Subjects	Exact Sign-Flip *p*	Noise-Free Gain (pp)
Frozen primary configuration	2.849 ± 1.712	9/9	0.0039	0.726
No reference-condition guard	3.429 ± 1.862	9/9	0.0039	0.859
Preservation weights 0.5×	2.879 ± 1.716	9/9	0.0039	0.587
Preservation weights 1.5×	2.869 ± 1.601	9/9	0.0039	0.814
Severe-noise weight 0.5×	2.559 ± 1.577	9/9	0.0039	0.883
Severe-noise weight 1.5×	2.934 ± 1.672	9/9	0.0039	0.651
Uniform SNR focus and selection	2.420 ± 1.542	9/9	0.0039	0.698

Note: Low-SNR gain averages −10, −5, and 0 dB within subject before group analysis; pp, percentage points; SD, standard deviation.

**Table A2:** Held-out noise-free reconstruction diagnostics.

*K*	NMSE, Mean ± SD	Pearson *r*, Mean ± SD	MSE Reduction vs. Channel-Mean Predictor (%)	Subjects with MSE Reduction
16	0.961 ± 0.040	0.171 ± 0.101	3.87 [0.83, 6.91]	8/9
32	0.960 ± 0.039	0.175 ± 0.112	4.01 [0.98, 7.04]	8/9
64	0.959 ± 0.036	0.189 ± 0.101	4.06 [1.32, 6.80]	8/9

Note: Values are from the 162 frozen checkpoints evaluated on held-out opposite-session trials without retraining; brackets are descriptive 95% confidence intervals. The channel-mean predictor was estimated from the training set. NMSE, normalized mean squared error; MSE, mean squared error; SD, standard deviation.

**Table A3:** Filter-bank EEGNet minus FBCSP–PCA balanced accuracy (percentage points).

*K*	−10 dB	−5 dB	0 dB	5 dB	10 dB	15 dB	20 dB	NF
16	2.06	1.43	−2.04	−5.25	−6.89	−7.46	−7.52	−7.58
32	3.34	1.19	−3.70	−6.85	−8.52	−9.01	−9.29	−9.44
64	4.22	0.11	−4.82	−7.66	−9.11	−9.55	−9.71	−9.77

Note: The learned transmitter and FBCSP–PCA reference used identical held-out trials, message dimensions, channel seeds, SNR conditions, and realization counts; NF, noise-free. No positive low-SNR comparison survived exact 24-condition max-|t| correction.

**Table A4:** Single-thread CPU forward-path complexity.

Model Path	*K*	Parameters	MACs/Trial	Median Latency, ms (5th–95th Percentile)
Common receiver	16	1348	1280	0.0384 (0.0379–0.0498)
Semantic residual + receiver	16	4564	4352	0.1594 (0.1542–0.1954)
Reconstruction encoder + receiver	16	67,700	7,553,792	1.2522 (1.2274–1.7351)
Filter-bank EEGNet + receiver	16	177,996	32,548,416	8.7065 (7.6580–14.3936)
Common receiver	32	2372	2304	0.0381 (0.0377–0.0460)
Semantic residual + receiver	32	8708	8448	0.1577 (0.1519–0.3077)
Reconstruction encoder + receiver	32	76,932	7,563,008	1.2762 (1.2490–1.8542)
Filter-bank EEGNet + receiver	32	183,132	32,553,536	7.8090 (7.5127–14.2472)
Common receiver	64	4420	4352	0.0386 (0.0380–0.0710)
Semantic residual + receiver	64	16,996	16,640	0.1572 (0.1545–0.1873)
Reconstruction encoder + receiver	64	95,396	7,581,440	1.2899 (1.2559–2.7675)
Filter-bank EEGNet + receiver	64	193,404	32,563,776	7.7817 (7.5841–9.4390)

Note: Batch size one, one CPU thread, 100 warm-up iterations, and 1000 timed iterations. FBCSP–PCA extraction, filter-bank processing, and common signal preprocessing were excluded; these are model-path rather than end-to-end timings. Parameter counts cover the stated inference path and exclude the unused reconstruction decoder. CPU, central processing unit; MAC, multiply–accumulate.
